# Comparative analysis of the *Oenococcus oeni* pan genome reveals genetic diversity in industrially-relevant pathways

**DOI:** 10.1186/1471-2164-13-373

**Published:** 2012-08-03

**Authors:** Anthony R Borneman, Jane M McCarthy, Paul J Chambers, Eveline J Bartowsky

**Affiliations:** 1 The Australian Wine Research Institute, PO Box 197, Glen Osmond, South Australia 5064, Australia

**Keywords:** Comparative genomics, Oenococcus, Industrial microbiology

## Abstract

**Background:**

*Oenococcus oeni,* a member of the lactic acid bacteria, is one of a limited number of microorganisms that not only survive, but actively proliferate in wine. It is also unusual as, unlike the majority of bacteria present in wine, it is beneficial to wine quality rather than causing spoilage. These benefits are realised primarily through catalysing malolactic fermentation, but also through imparting other positive sensory properties. However, many of these industrially-important secondary attributes have been shown to be strain-dependent and their genetic basis it yet to be determined.

**Results:**

In order to investigate the scale and scope of genetic variation in *O. oeni*, we have performed whole-genome sequencing on eleven strains of this bacterium, bringing the total number of strains for which genome sequences are available to fourteen. While any single strain of *O. oeni* was shown to contain around 1800 protein-coding genes, in-depth comparative annotation based on genomic synteny and protein orthology identified over 2800 orthologous open reading frames that comprise the pan genome of this species, and less than 1200 genes that make up the conserved genomic core present in all of the strains. The expansion of the pan genome relative to the coding potential of individual strains was shown to be due to the varied presence and location of multiple distinct bacteriophage sequences and also in various metabolic functions with potential impacts on the industrial performance of this species, including cell wall exopolysaccharide biosynthesis, sugar transport and utilisation and amino acid biosynthesis.

**Conclusions:**

By providing a large cohort of sequenced strains, this study provides a broad insight into the genetic variation present within *O. oeni*. This data is vital to understanding and harnessing the phenotypic variation present in this economically-important species.

## Background

Like many fermented foods and beverages, wine represents a historical method of nutrient preservation that relies on suppressing the growth of spoilage microorganisms in order to provide long-term storage. In finished wine, it is the physiochemical combination of high levels of ethanol and sulfur dioxide, scarcity of “preferred” nutrient sources and low pH that combine to produce a harsh environment in which all but a small number of microorganisms can proliferate.

*Oenococcus oeni,* a member of the lactic acid bacteria (LAB), is one of the limited number of bacterial species that not only survive, but actively grow in wine. In fact, *O. oeni* is present at extremely-low to undetectable levels on intact grapes or in the general environment, with wine seemingly representing the exclusive niche of this bacterium, despite the seasonal nature of wine production [1-3]. It is therefore fortunate that *O. oeni* provides positive attributes with regard to wine quality, as the majority of other wine-associated bacterial species are linked with spoilage [4]. The primary recognised role of *O. oeni* in winemaking is in performing malolactic fermentation (MLF), a de-acidification reaction in which malic acid is decarboxylated to lactic acid [5]. However, in addition to performing MLF, there is evidence that the growth of *O. oeni* in wine impacts on flavor, aroma and mouth-feel in a strain-specific manner [6-8].

Recently, the genome sequences of three *O. oeni* strains, PSU-1 [9,10], BAA-1163 and AWRIB429 [11] were released. Genomic comparisons indicated that, for a free-living microorganism, *O. oeni* has a compact genome of approximately 1.8 Mb, which presumably reflects genomic streamlining that has occurred during its adaptation to the ecologically-restricted niche of fermenting grape juice and wine [10,12,13]. However, despite its already streamlined genome the three strains of *O. oeni* were shown to display significant inter-strain genomic variation with the potential for up to 10% variation in protein coding genes [11].

To define the scale and scope of the pan and core genomic potential of this important industrial species, we have sequenced a further eleven strains of *O. oeni* from both commercial and environmental sources, bringing the total number of available genomes for this species to fourteen. Comparison of this expanded group of isolates has identified additional variation across the species, defined clear multi-strain groups with conserved sets of strain-specific genes and genomic deletions, and has provided genetic bases for phenotypic characteristics that separate specific strains.

## Results and discussion

The genome sequences for eleven strains of *O. oeni* were each assembled from 1 x 10^6^ Illumina sequencing reads (100 bp, paired-end library) using MIRA. Of the eleven strains, six were chosen based upon diverse microarray-based comparative genome hybridization profiles [11], while the remainder were selected as Australian environmental isolates acquired over five decades in addition to the official type strain of *O. oeni* (DSM2052; AWRIB129). Manual curation of each assembly resulted in the genomic sequence of each strain being captured in a small number of single-copy contigs separated by low-copy number repeats whose length was greater than that used for the paired-end library construction (~500 bp) (Table1). Using conserved genomic synteny, these contigs were then rationally assembled into chromosomal super-contigs through positioning these repeat sequences in each of their multiple genomic locations (See Additional file 1). This resulted in near complete chromosomal assemblies for each strain (average 2.36 assembly gaps per strain), providing a solid foundation for inter-strain comparison (Table1). 

**Table 1:** Assembly statistics

**Strain**	**Total contigs**	**Repeat contigs**	**Chromosomal super contigs**	**Plasmids**	**Unplaced contigs (scaffolds)**
AWRIB129	42	13	1	0	0
AWRIB202	36	9	3	0	0
AWRIB304	38	9	4	0	6 (1)
AWRIB318	26	7	1	0	0
AWRIB418	34	10	4	0	0
AWRIB419	46	13	4	1	0
AWRIB422	32	7	6	1	0
AWRIB548	29	8	2	0	0
AWRIB553	32	9	1	0	0
AWRIB568	31	7	6	1	0
AWRIB576	28	7	3	1	0

In addition to chromosomal-associated nucleotide sequences, there were small circular replicons that were identified in at least four strains (AWRIB419, AWRIB422, AWRIB568 and AWRIB578). While this plasmid was of similar size across the four strains and often encoded many proteins with similar predicted functions (Table1), there appears to be at least three distinct types. In each case, the plasmid appears to encode only those functions required for replication and maintenance, with the exception of a putative glycerate dehydrogenase (2-hydroxyacid dehydrogenase) found in plasmids from AWRIB422, AWRIB568 and AWRIB576 and a putative NADH:flavin oxidoreductase found in plasmids from all four strains. At this point, the selective advantage (if any) provided by these plasmids and these enzymatic functions remains to be determined.

### Pan and core genomes of *O. oeni*

Previous comparative genomic research was able to show that there was significant genomic variability between three *O. oeni* isolates for which genome sequence information was available prior to this study [9-11,14]. In order to examine the extent of protein-coding variability across this expanded set of fourteen strains, open reading frames (ORFs) were predicted from each genomic dataset (See Methods) and this information was used to produce detailed genomic orthology comparisons that were based upon sequence homology combined with genomic synteny (See Additional file 2). As a basis for orthology mapping, the original genomic annotation of *O. oeni* strain PSU-1 was used as a reference annotation as this was both the first *O. oeni* strain to be sequenced and is also the only strain for which a complete, finished genome sequence is available [9,10].

**Figure 1:**
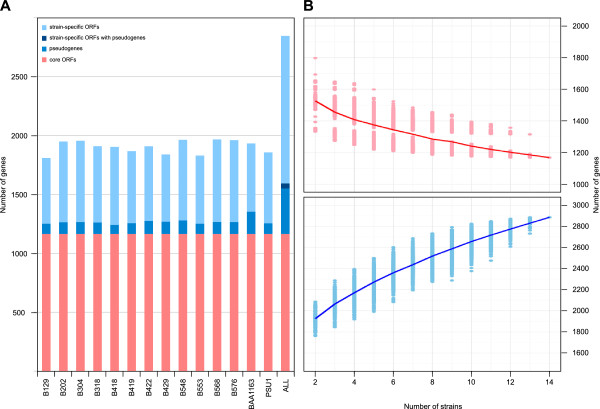
**Core and pan genomes of *O. oeni *. A.** Proportions of core (conserved) ORFs, strain-specific ORFs and putative pseudogenes in fourteen strains of *O. oeni*. The overall number of each type of ORF is also indicated based upon syntenic assignment of orthologs across all fourteen strains. **B.** The estimated average size of the *O. oeni* core (pink solid line) and pan (light blue solid line) genomes as a function of the number of individual strains compared. For each point, the size of core and pan genome was calculated for all combinations of *x* strains from the fourteen strains analysed with the results presented as filled circles.

On average, each strain was predicted to contain 1800 ± 52 full length ORFs and 104 ± 27 potential pseudogenes associated with the chromosomal replicon (Figure1), with some strains also containing ORFs (n<50) associated with the plasmid replicon. However, despite the relatively tight distribution in the number of protein-coding genes in each strain, there is considerable variation in the subset of ortholgous genes present in each strain. In order to quantify this variation in coding potential, the extent of the core and pan genomes of this collection of *O. oeni* strains were calculated. There were 2846 non-degenerate ORFs that were shown to comprise the chromosomal pan genome of this group of *O. oeni* strains, with 1165 of these representing core ORFs conserved across all fourteen strains (Figure1). As observed for other bacterial species, the size of the conserved core of protein from *O. oeni* decreases as a function of the number of strains compared, while size of the pan genome increases [15-17] (Figure1B). Also, given that the rate of expansion of the pan genome showed no signs of significant decrease as additional numbers of strains were added to the analysis, it appears that the genetic diversity present within this strain has not yet been exhaustively recorded.

In total, there were 1064 non-degenerate ORFs that were predicted to encode full-length functional proteins (based on homology searches) in at least one of the strains (in addition to 93 pseudogenes) that are absent in the original PSU-1 gene annotation [9,10] (Additional file 3). Of these, 64 are due to annotation differences and are found in the PSU-1 genome via the annotation pipeline applied in this study compared to that used in the original annotation of the PSU-1 genome. For an additional 58 of these, pseudogenes exist in PSU-1 whereas full-length proteins are present in at least one other strain. The remaining 942 ORFs are the result of strain-specific insertion events in strains other than PSU-1. In addition, over one third (348) of these non-PSU-1 proteins display their closest homology to proteins from outside of *O. oeni* (including protein-coding genes from BAA-1163 and AWRIB429) and represent new additions to the *O. oeni* pan genome.

### Horizontal gene transfer

**Figure 2:**
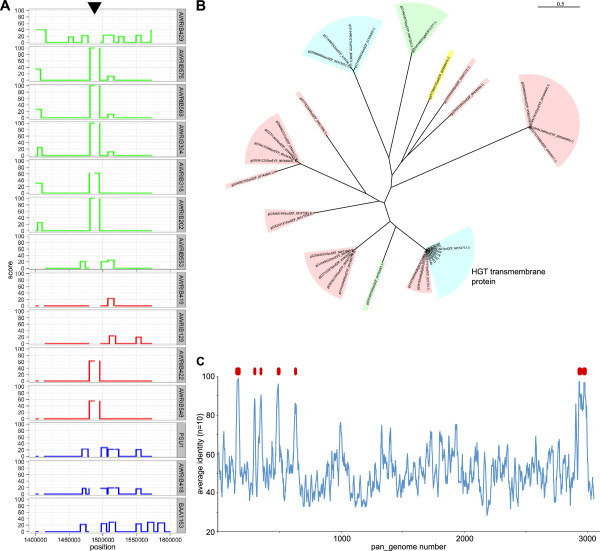
**Horizontal gene transfer in *O. oeni*. A.** The probability of genomic regions being present due to horizontal gene transfer (HGT). A region of high probability of strain-specific HGT is indicated (black arrow). **B.** Maximum-likelyhood phylogeny of a horizontally-acquired transmembrane protein within the region highlighted in **(A)**. Bacterial genera are indicated by colored shading (*Oenococcus spp.*, blue; *Lactobacillus spp.*, pink; *Weissella spp.*, green; *Pediococcus spp.*, yellow). **C.** Homology between *O. oeni* and *Lactococcus spp*. A representative protein for each pan_genome locus was used in homology searches against the combined predicted protein sequences from each *Lactococcus spp*. genome available in Genbank. Average identity values were calculated using a 10 ORF sliding window (blue line). Regions predicted to be result of HGT in which >5 adjacent ORFs displayed >90% amino acid identity between an *O. oeni* protein and a protein from *Lactococcus spp*. are also indicated (red boxes).

In order to determine if any of the strain-specific genes in the *O. oeni* genome were the result of horizontal gene transfer (HGT), the genome of each strain was interrogated for regions with an increased probability of being horizontally-acquired [18]. While there were numerous regions that exceeded the threshold for being potentially horizontally acquired (Additional file 4), one region, present in at least seven of the strains, had a very high probability of resulting from HGT , (Figure2A). This region was subsequently shown to contain evidence of two independent HGT events (separated by ~65 kb) involving IS element insertions that appear to have been horizontally transferred from *Lactobacillus spp*. The first of these (pan_genome loci 1725–1734) appears to be associated with an IS30 element insertion that was first discovered in AWRIB429 [11,19]. The second region (pan_genome loci 1802–1816) is associated with an insertion event within an ORF that encodes an arginine deaminase (OEOE_1118 of PSU-1). The last 3.5 kb of this 7.5 kb fragment has ~99% identity to a large portion of a genetic element that has been characterised in beer-spoilage strains of *Lactobacillus spp*. and *Pedicoccus spp*. [20]. This element has been postulated to be horizontally-transferred and, due to the presence of the HorC efflux pump, to provide resistance to antibacterial compounds present in hops [21]. However, the portion of the element present in strains of *O. oeni* lacks the *horC* gene, while still encoding the glycosyltransferase, an integral membrane protein, and a cell wall teichoic acid glycosylation protein that are present in the 3’ half of the element. As expected for a HGT event, these proteins share a higher than expected degree of relatedness compared to the evolutionary distance that separates *Oenococcus spp.* and *Lactobacillus spp.* (Figure2B, Additional file 2).

There were at least another five genomic insertions in various subsets of the strains (including regions within the extra-chromosomal replicons) that appear to be the result of HGT. Based on homology searches, the HGT events giving rise to these elements, like those mentioned previously, appear to have originated from *Lactobacillus spp*. (Figure2C, Additional file 5). It therefore appears that *Lactobacilli* provide a potential reservoir of genes for *O. oeni*.

### Highly variable bacteriophage integration across strains

**Figure 3:**
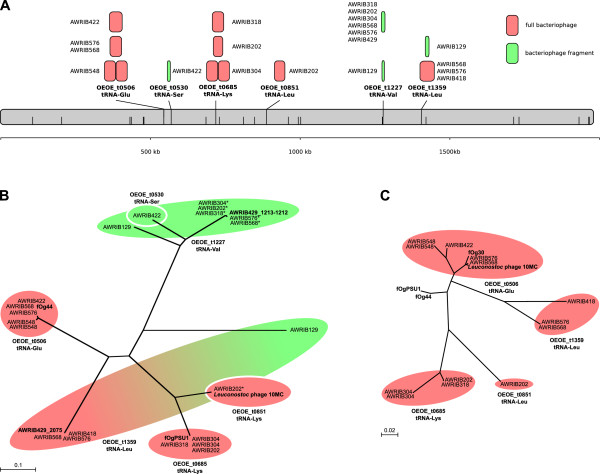
**Bacteriophage diversity. A.** Sites of bacteriophage integration throughout the *O. oeni *genome are indicated by along with the name of the specific tRNA that represents the bacteriophage attachment site. Other tRNA genes predicted to be present in the *O. oeni* genome are also indicated (half-height black lines). Full bacteriophage elements (pink boxes) are characterised by the presence of both integrase (*int*) and lytic *(lys)* enzymes while bacteriophage fragments (green boxes) generally only contain *int* ORFs. All elements are drawn to scale. **B.** Maximum-likelyhood phylogenetic analysis of bacteriophage *int* proteins from *O. oeni*. Colored shading is used to group proteins based on their site of integration and to define their origin as either being from a full bacteriophage element (pink) or a bacteriophage fragment (green). Previously identified phage protein sequences are indicated in bold. **C.** Maximum-likelyhood phylogenetic analysis of bacteriophage *lys* proteins from *O. oeni*. Shading is identical to that in part B.

One of the most striking variations in intra-specific coding potential across the *O. oeni* strains was in the number and position of temperate bacteriophage integrations (Figure3). *O. oeni* has been shown previously to harbour at least four separate bacteriophages that integrate through tRNA-associated attachment sites (fOgPSU1, fOg44, fOg30 and Φ10MC) [22-25]. In this study, a total of six different tRNAs were shown to potentially be involved in the integration of bacteriophage, with four shown to be current sites of insertion of full-length and presumably functional bacteriophage (Figure3A). The remaining two tRNAs (plus a third in AWRIB129) contained bacteriophage remnants and may represent sites at which integration and then subsequent excision of a bacteriophage has occurred. It was also apparent that for two of the insertion events (at OEOE_t0506 in AWRIB548 and OEOE_t685 in AWRIB304), the entire bacteriophage sequence had been tandemly duplicated at the integration site.

In order to determine phylogenetic relationships between bacteriophage sequences, genes encoding the highly conserved bacteriophage integrase (*int*) and endolysin (*lyt*) proteins (if present) were used to construct phylogenies for each integration event. For the integrase proteins (Figure3B), it was possible to show that most proteins grouped according to the particular tRNA at which the integration occurred, with the exception of two *int* ORFs from the bacteriophage fragments in AWRIB129; the latter was significantly different from the other *int* ORFs present at the same tRNA site. This linking of tRNA and *int* sequence also extended to the known sequences of fOgPSU1, fOg44 and Φ10MC that were found to group according to their previously identified tRNA sites [23,25]. However, this linking between site of tRNA integration and int sequence did not hold for the lys genes (Figure3C), where there was considerable variation in the sequence of the endolysins encoded by the ORFs found at each tRNA sequence between the previously known bacteriophage elements and those found in this study.

### Phylogenetic diversity

**Figure 4:**
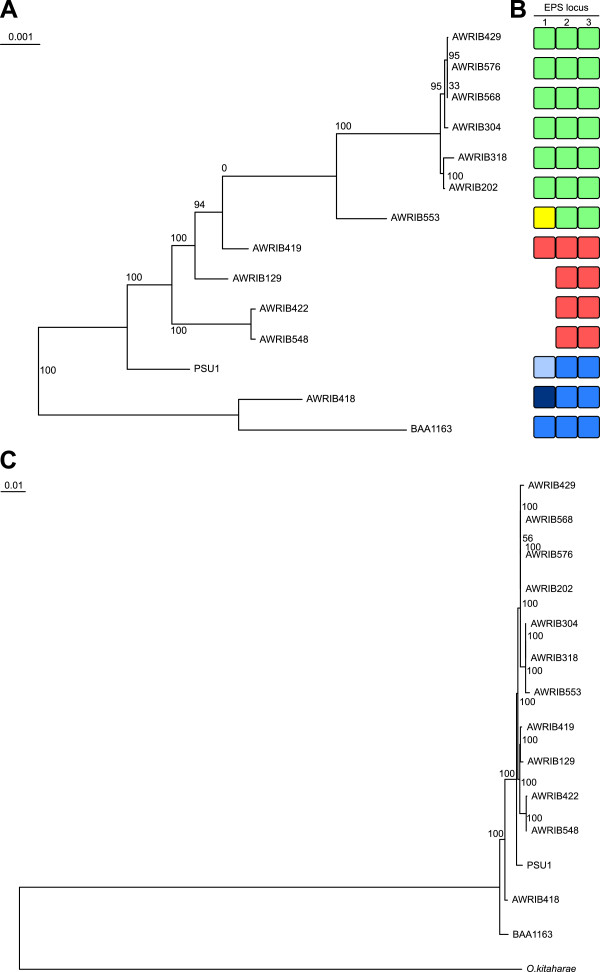
**Phylogenetic divergence of *O. oeni*. A.** Maximum-likelyhood phylogeny based on whole-genome alignment of fourteen *O. oeni* strains. Bootstrap proportions are indicated at each relevant position (total support from 100 replicates). **B.** Variation in exopolysaccharide (EPS) loci in *O. oeni*. Each of the three loci is composed of a variable number of individual ORFs. Each variant at a particular locus is represented by a separate color. **C.** Maximum-likelyhood phylogeny based on the concatenated predicted protein sequences of 872 conserved orthologs from fourteen strains of *O. oeni* in addition to its closest known relative, *O. kitaharae*[27].

Independent of coding-region predictions, it was possible to determine the phylogenetic relationship of the various strains from the patterns of single-nucleotide polymorphisms deduced from whole-genome nucleotide alignments (Figure4A). The phylogeny produced from this alignment led to two major findings. First there was a large evolutionary distance between a basal clade formed by AWRIB418 and BAA-1163 and the other twelve strains of *O. oeni,* suggesting that these two strains form a distinct evolutionary group, a finding that is supported by the results of previous MLST typing of typing [26]. In order to investigate this apparent division, a second phylogeny was constructed using the predicted sequences of the core, conserved proteins present in the fourteen *O. oeni* strains, in addition to orthologous sequences from *O. kitaharae* DSM 17330 as an outgroup [27] (Figure4C). This phylogeny is consistent with BAA-1163 and AWRIB418 comprising a basal, divergent clade, however the genetic distance between these groups of strains is far less than observed between any strain of *O. oeni* and *O. kitaharae*. As such, BAA-1163 and AWRIB418 may together represent a divergent sub-species of *O. oeni*, a fact that is supported by the presence of a large number of ORFs that are found only in these two strains (See below).

The second finding from the *O. oeni* strain phylogeny, is the very close genetic relationship between the strains AWRIB429, AWRIB576, AWRIB568, AWRIB304, AWRIB318 and AWRIB202. Interestingly, all of these strains, with the exception of AWRIB429 (Lalvin VP41) which was isolated in Italy, was isolated from natural wine ferments in Australia over a fifty year period (Table2) (Figure4A). Given the unexpected nature of the phylogenetic relationship between the Australian isolates and AWRIB429, the identity of AWRIB429 as an isolate of VP41 was confirmed by sequencing one of the cell wall loci (locus 2) from a second, independent isolate of VP41 which was obtained from the supplier of this strain (AWRIB551). This second isolate has a nucleotide sequence that was 100% identical to that of AWRIB429, indicating that these two strains most likely both represent Lalvin VP41 and that the observed phylogeny is in fact a true representation. However the cause of this relationship between VP41 and the Australian isolates remains to be determined, but it is interesting to note that the isolation of many of the Australian strains predates the use of Lalvin VP41 as a commercial malolactic starter strain, therefore environmental introduction of this strain through winery inoculation can be all but excluded as a hypothesis.

**Table 2:** Strains used in this study

**Strain**	**Other isolate name(s)**	**Commercial supplier**	**Place of origin**	**Reference(s**	**Genbank accession number**
AWRIB129	Type strain; DSM 20252		France	[28]; This study	AJTP00000000
AWRIB697	BAA-1163; IOB8413				AAUV00000000
AWRIB563	PSU-1		USA (1972)	[29]	NC_008528
AWRIB429	Lalvin VP41	Lallemand	Abruzzi region, Italy	[11]; This study	ACSE00000000
AWRIB418	MCW	Lallemand	Sonoma Country, USA	This study	ALAE00000000
AWRIB419	Lalvin EQ54	Lallemand	Cote de Rhone, France	This study	ALAF00000000
AWRIB553	Elios-1	Lallemand	Tuchan Languedoc, France	This study	ALAI00000000
AWRIB548	MT01; BL01	Lallemand	France	This study	ALAH00000000
AWRIB422	Viniflora CH35	Chr Hansen		This study	ALAG00000000
AWRIB202			Coonawarra, Australia (1994)	[11]; This study	AJTO00000000
AWRIB304			Adelaide Hills, Australia (1999)	[11]; This study	AJIJ00000000
AWRIB318	NCDO 1884		Merbein, Australia (1956/57)	[30]; This study	ALAD00000000
AWRIB568			Mt Mary, Australia (2008)	This study	ALAJ00000000
AWRIB576			Mt Mary, Australia (2008)	This study	ALAK00000000

### Cell wall exopolysaccharide variation

Using whole-genome sequence data, it was shown previously that significant differences existed in the composition of the exopolysaccharide (EPS) operons of *O.oeni* PSU-1, BAA-1163 and AWRIB429 [11,31]. In the current expanded study, it was possible to define three separate clusters of ORFs that are potentially associated with EPS production and that show substantial variation across the fourteen strains (Figure4B; Additional file 6). At each of the three loci, there was a variable number of genomic “cassettes” (locus 1 has 7 different genetic cassettes, locus 2, 3 cassettes and locus 3, three cassettes) providing the basis for a high degree of potential intraspecific diversity in the composition of *O. oeni* cell wall.

The combination of independent cell wall loci in each strain was consistent with the whole-genome phylogenies and defined three main groups of strains (Figure4B; Additional file 6). It is predicted that these groups therefore comprise three distinct cell wall types that approximate serotype variation that is observed in some species of pathogenic bacteria [32]. Also, as bacterial EPS can potentially interact with, and therefore modulate components of wine, there is the potential for these different EPS genotypes to differentially affect wine quality. This is consistent with reported differences in both the amount and type of EPS produced by at least three of the strains in this study that contain different EPS operon combinations (PSU-1 BAA-1163 and AWRIB429) [31].

### Sugar transport and utilisation

**Figure 5:**
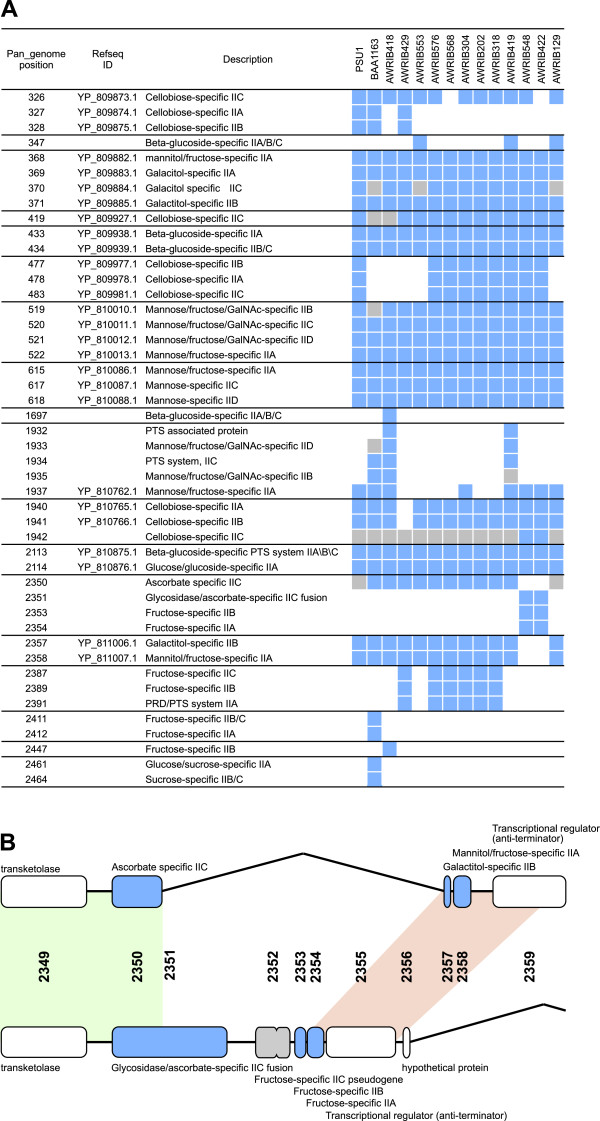
**Variation in phosphotransferase (PTS) enzyme II systems in *O. oeni*. A.** The occurrence and location of PTS enzyme II clusters across fourteen strains of *O. oeni*. The presence of a particular ORF in a specific strain is indicated (blue shading). Pseudogenes are also shown (grey shading). Individual PTS clusters are grouped by solid lines. **B.** A novel PTS IIC-glycosidase gene fusion in *O. oeni* strains AWRIB422 and AWRIB548. The two alternative genotypic arrangements are shown with areas of near complete sequence homology indicated by green shading. Genomic loci that display functional similarity (e.g. both proteins are PTS IIA subunits) are also indicated by pink shading.

The range of sugars that *O. oeni* is capable of utilising is strain dependent [33]. To investigate the genetic basis of differences in sugar metabolism, we began by classifying genes that are predicted to encode phosphotransferase system (PTS) enzyme II sugar transporters in the *O. oeni* pan genome. In total, there were 46 PTS subunits that grouped into 18 separate genomic loci (individual subunits being adjacent in the genome) (Figure5). Fourteen of these loci contained either individual or multi-domain ORFs encoding IIA, IIB and IIC functions (and in two cases IID) in at least one strain and would therefore be expected to encode fully functional transporter complexes (Figure5). Correlating with differences in carbohydrate utilisation, only three of these complexes were conserved across all fourteen strains (in addition to three others in which strains contained potential pseudogenes in otherwise complete complexes).

Interestingly, for one specific PTS cluster (present in AWRIB548 and AWRIB422), there appears to have been a strain-dependent genomic insertion event that resulted in the incorporation of two different IIA, IIB duplexes with a conserved IIC subunit (pan_genome loci 2350 to 2358) (Figure5B). Accompanying this exchange of IIB and IIC subunits, this genomic insertion event has resulted in the formation of a fused ORF encoding the conserved PTS IIC subunit at the 3' end and a sequence that encodes a protein with high homology to glycosidases at the 5' end. There have been many instances of PTS subunit protein fusions reported previously [34]. However, the PTS IIC-glycosidase fusion found in AWRIB548 and AWRIB422 appears to be novel, as homology searches provide significant matches to both the amino- and carboxyl- sections of the fusion protein in isolation, but lack single protein matches across the entire length of the predicted fusion protein (Additional file 7). While the function of this protein remains to be determined, it is tempting to speculate that the glycosidase domain of the fusion can catalyse the release of sugars that can be subsequently transported into the cell via the PTS system.

**Figure 6:**
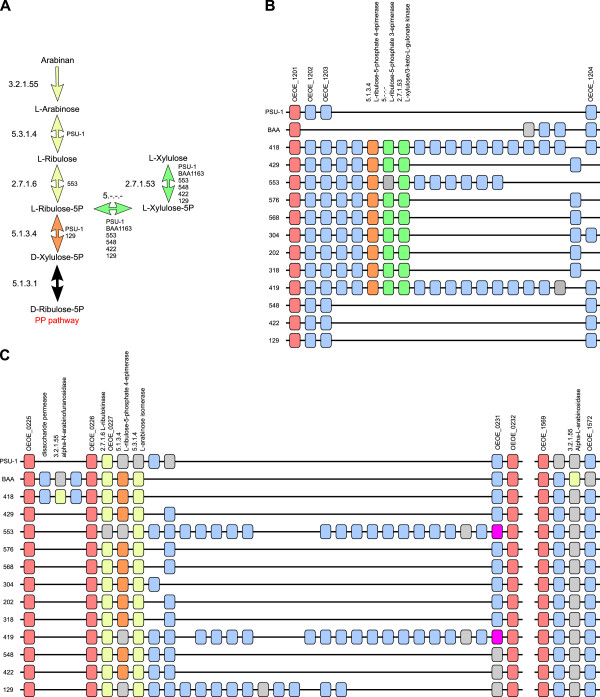
**Variations in sugar utilisation pathways in *O. oeni*. A.** The predicted pathway for the assimilation of L-arabinose and L-xylulose in *O. oeni*. EC numbers are provided for each enzymatic step. *O. oeni* strains displaying metabolic blocks at individual enzymatic steps are also indicated. Arrows are color-coded according to the location of the ORF in parts B and C. **B.** A strain-specific genomic insertion predicted to impart the ability to utilise L-xylulose. Individual ORFs surrounding this locus are shown for each strain. Orthologous ORFs are positioned vertically and are color coded according to their conservation status (conserved, pink; strain-specific, light-blue). ORFs predicted to be directly involved in the utilisation of L-xylulose are color-coded according to their metabolic role (green, L-xylulose utilisation, orange, L-xylulose and L-arabinose utlisation). **C.** A strain-specific genomic insertion predicted to impart the ability to utilise arabinan and L-arabinose. Shading is identical to part B except for the presence of ORFs involved directly in the utilisation of arabinan and L-arabinose (shaded yellow).

In addition to intra-specific variation in the PTS transporter systems of *O. oeni*, there were also differences in metabolic pathways for sugar utilisation (Figure6). Arabinose and xylose are two sugars that have been noted as displaying strain-dependent utilisation profiles in *O. oeni*[33]. While genes imparting the ability to utilise xylose were not evident in the sequenced strains, the data supports strain-dependent metabolic potential to utilise L-arabinose (including the arabinose polymer arabinan) and L-xylulose (Figure6A).

The potential to utilise L-xylulose is predicted to be dependent on an insertion event found in nine of the fourteen strains, which introduces the three enzymes predicted to be required for conversion of L-xylulose to D-xylulose-5-phosphate (for entry in the pentose phosphate pathway) (Figure6B). L-arabinose utilisation is also encoded by a set of three enzymes. However, unlike the L-xylulose cluster, the genomic locus containing the arabinose metabolism associated ORFs was found to be present in all fourteen strains, with the intraspecific utilisation of L-arabinose being predicted to be due to the presence of nonsense mutations in one or more of the three ORFs in some strains (Figure6C). In addition to the ability to utilise L-arabinose, BAA-1163 and AWRIB418 are predicted to be capable of degrading the arabinose polymer arabinan (Figure6A). Interestingly, this ability in AWRIB418 is predicted to be due an insertion event specific to this strain and BAA-1163, however the ORF in BAA-1163 ORF is interrupted by a nonsense mutation. In contrast, BAA-1163 is predicted to utilise arabinan through the function of a conserved genomic locus that is predicted to be a pseudogene in all but this strain (Figure6C).

In addition to their ability to utilise arabinan, AWRIB418 and BAA-1163 also encode a functional α-glucosidase (pan genome ORF 1409; pseudogene in the other strains), which is predicted to allow these strains to utilise sucrose (a rare-trait in *O. oeni*[33]), while the BAA-1163 genome is also predicted to encode a L-iditol 2-dehydrogenase (pan genome ORF 2413; strain-specific insertion) that would allow for the utilisation of D-sorbitol through its conversion to D-fructose. Interestingly, while the utilisation of fructose by *O. oeni* is a ubiquitous property of these strains [33], the metabolic pathway for the catabolism of this sugar is lacking in the PSU-1 genome due to a frameshift in the coding region of the gene encoding fructose-bisphosphate aldolase (4.2.1.14). The full-length coding region is present in all other sequenced strains of O. oeni and this information, combined with the phenotypic data available for *O. oeni*, would suggest that this difference likely represents a sequencing error in the current PSU-1 sequence.

### Amino acid biosynthesis

*O. oeni* has a variety of amino acid auxotrophies with several showing intraspecific differences [28]. Initial analysis of the genome of *O. oeni* PSU1 suggested the presence of biosynthetic pathways for up to eight amino acids [10]. In the expanded dataset provided by the fourteen genome sequences generated in this study, it is apparent that there are instances of intra-specific diversity in the ability to synthesise specific amino acids (Table3). One of these main differences is in the predicted ability of the divergent *O. oeni* strains BAA-1163 and AWRIB418 to potentially synthesise the amino acid leucine. While most strains of *O. oeni* lack the enzyme 3-isopropylmalate dehydrogenase (EC:1.1.1.85) due to the presence of a conserved non-sense mutation in the ORF, both BAA-1163 and AWRIB418 are predicted to encode a full-length functional enzyme (Additional file 1, pan genome ORF 2716). In addition to this potential difference in leucine auxotrophy, there are predicted differences in the ability to synthesize threonine, glutamine and methionine due to the presence of nonsense mutations in genes involved in these pathways in specific strains (Table3). The findings for these amino acids are consistent with previous phenotypic tests that found variable responses for these amino acids in this species [28]. 

**Table 3:** Strain-dependent differences in amino acid genes

**Amino acid**	**Enzyme**	**Pan_genome number**	**Strain(s) affected**
L-threonine	homoserine kinase	2510	AWRIB304
	2.7.1.39		PSU-1
L-glutamine	L-glutamate synthetase	1612	AWRIB202
	6.3.1.2		
L-methionine	Aspartate kinase	2507	AWRIB304
	2.7.2.4		AWRIB422
			AWRIB548
	O-succinylhomoserine (thiol)-lyase	1350	PSU-1
	2.5.1.48		AWRIB129
			AWRIB202
			AWRIB304
			AWRIB419
			AWRIB422
			AWRIB429
			AWRIB553
			AWRIB568
			AWRIB576
	Methionine synthase II	1895	BAA-1163
	2.1.1.14		AWRIB202
			AWRIB304
			AWRIB318
			AWRIB422
			AWRIB429
			AWRIB548
			AWRIB568
			AWRIB576
L-leucine	3-isopropylmalate dehydrogenase	2716	PSU-1
	1.1.1.85		AWRIB129
			AWRIB202
			AWRIB304
			AWRIB318
			AWRIB419
			AWRIB422
			AWRIB429
			AWRIB548
			AWRIB553
			AWRIB568
			AWRIB576

## Conclusions

Like other industrial species, of microorganisms phenotypic variation in *O. oeni* will have direct economic consequences through impacts on product quality and production efficiencies. A thorough understanding of the basis of this variation therefore provides the means to improve the industrial performance of these strains or to easily screen for new strains with multiple, desirable traits. This study provides a solid foundation for the investigation of phenotypic diversity in *O. oeni* by providing whole-genome sequences for a large cohort of strains from both commercial and environmental sources. As such, we have identified significant variation across the strains that were investigated, including differences in cell wall synthesis and sugar utilisation, that were largely due to differential insertion of large, multi-genic nucleotide fragments. These differences can be used to inform research on the industrial implications of this genetic variation while allowing for the identification of strains with combinations of desirable genetic, and therefore phenotypic, characteristics.

## Methods

### Strains and growth conditions

Strains used in this study are listed in Table2 and were selected to represent a cross-section of commonly used commercial strains, in addition to Australian environmental isolates present in the AWRI culture collection. To prepare genomic DNA, each strain was grown in MRS (Amyl Media, Australia) supplemented with 20% apple juice [35] for between six and ten days at 27°C. DNA was prepared by phenol chloroform extraction as previously described [36].

### Genome sequencing and assembly

Genome sequencing was performed at the Ramaciotti Centre for Gene Function Analysis (University of New South Wales, NSW, Australia) using Illumina sequencing technology and 2 x 100 bp paired-end sequencing reads. For each strain 1 x 10^6^ individual reads were *de novo* assembled using MIRA v 3.2.1 and manually refined using Seqman Pro (DNAstar). These Whole Genome Shotgun projects, including ORF predictions, have been deposited at DDBJ/EMBL/GenBank with the appropriate accession number for each strain used in this study listed in Table2.

### Genome annotation and comparison

Potential coding regions were predicted using Glimmer [37]. Genomic orthology was assigned via reciprocal homology searches using BLAST [38] combined with long-range genomic synteny. Intra-specific whole genome alignments were produced using FSA [39] and used to construct a maximum-likelyhood phylogeny using PhyML [40]. For comparison to *O. kitaharae*, conserved protein orthologs were identified via homology (minimum 60% identity when compared to the homologous *O. kitaharae* protein) in all of the *O. oeni* genomes used in this study. Next, proteins which had potential paralogs (which could confound the phylogeny) were identified by assigning each protein to specific orthoMCL [41] clusters and then only retaining those groups of orthologs in which each protein was the only member of a particular orthoMCL group. Individual protein alignments were then performed on each set of homologous sequences using Muscle [42]. These individual alignments were then concatenated into a single large sequence for each strain which was used to construct a maximum-likelihood phylogenetic tree using PhyML [40]. Bacteriophage phylogenies were produced by aligning individual predicted protein sequences using Muscle [42] and then producing maximum-likelyhood trees using PhyML [40]. Enzyme function annotations were made using the KAAS annotator via the KEGG website [43]. Regions of potential horizontal gene transfer were detected using alien hunter using HMM-derived change point detection [18].

## Competing interests

The authors declare that they have no competing interests.

## Authors’ contributions

A.R.B designed experiments, assembled and analysed the genomic data and prepared the manuscript. J.M.M. performed experimental work. P.J.C designed experiments and helped prepare the manuscript. E.J.B designed experiments and helped prepare the manuscript. All authors read and approved the final manuscript.

## Supplementary Material

Additional file 1 **Super contig structure.** Describes the position of each sequencing contig within the chromosomal super contigs in each strain. Click here for file

Additional file 2 **Syntenic alignment and annotation of *O. oeni* open reading frames.** Contains information regarding orthology relationships and gene function for open reading frames from each *O. oeni* strain for which sequencing data is available. Click here for file

Additional file 3 **Non-PSU-1 open reading frames.** Contains full positional and orthology information for all of the open reading frames not found in the “reference” strain PSU-1. Click here for file

Additional file 4 **Genome-wide results of the “alien hunter” algorithm for each strain.** Each individual plot has been spaced according to the whole-genome alignments to ensure that genomic regions are concordant across strains. The area of highest probability of HGT is indicated (black arrow). Click here for file

Additional file 5 **Horizontal gene transfer events between *O. oeni* and *Lactococcus spp*.** A representative protein (query_id) for each pan_genome locus was used in homology searches against the combined predicted protein sequences from each *Lactococcus spp*. genome (seq_id) available in Genbank. Average identity values were calculated using a 10 ORF sliding window (average(n = 10)). Regions in which >5 adjacent ORFs displayed >90% amino acid identity between an *O. oeni* protein and a protein from *Lactococcus spp*. are also indicated (window n = 5; identity > = 90). Click here for file

Additional file 6 **Exopolysaccharide gene clusters.** Contains full positional and orthology information for all of the open reading frames predicted to encode members of the strain-dependent cell wall clusters. Click here for file

Additional file 7 **A novel glycosidase-PTS gene fusion.** BLAST homology information for the glycosidase-PTS gene fusion predicted to be encoded by pan_genome ORF 2351. (PDF 44 kb) Click here for file

## References

[B1] Bae S Fleet GH Heard GM Lactic acid bacteria associated with wine grapes from several Australian vineyards J Appl Microbiol 2006 100 712 727 10.1111/j.1365-2672.2006.02890.x 16553726

[B2] Renouf V Claisse O Lonvaud-Funel A Understanding the microbial ecosystem on the grape berry surface through numeration and identification of yeast and bacteria Aust J Grape Wine Res 2005 11 316 327 10.1111/j.1755-0238.2005.tb00031.x

[B3] Renouf V Claisse O Lonvaud-Funel A Inventory and monitoring of wine microbial consortia Appl Microbiol Biotechnol 2007 75 149 164 10.1007/s00253-006-0798-3 17235561

[B4] Bartowsky EJ Bacterial spoilage of wine and approaches to minimize it Lett Appl Microbiol 2009 48 149 156 10.1111/j.1472-765X.2008.02505.x 19141041

[B5] Henick-Kling T Fleet GH Malolactic fermentation Wine microbiology and biotechnology 1993 Chur, Switzerland: Harwood Academic Publishers 289 326

[B6] Bartowsky EJ Henschke PA The “buttery” attribute of wine–diacetyl–desirability, spoilage and beyond Int J Food Microbiol 2004 96 235 252 10.1016/j.ijfoodmicro.2004.05.013 15454314

[B7] Lonvaud-Funel A Lactic acid bacteria in the quality improvement and depreciation of wine Antonie Van Leeuwenhoek 1999 76 317 331 10.1023/A:1002088931106 10532386

[B8] Swiegers JH Bartowsky EJ Henschke PA Pretorius IS Yeast and bacterial modulation of wine aroma and flavour Aust J Grape Wine Res 2005 11 139 173 10.1111/j.1755-0238.2005.tb00285.x

[B9] Makarova K Slesarev A Wolf Y Sorokin A Mirkin B Koonin E Pavlov A Pavlova N Karamychev V Polouchine N Shakhova V Grigoriev I Lou Y Rohksar D Lucas S Huang K Goodstein DM Hawkins T Plengvidhya V Welker D Hughes J Goh Y Benson A Baldwin K Lee J-H Díaz-Muñiz I Dosti B Smeianov V Wechter W Barabote R Lorca G Altermann E Barrangou R Ganesan B Xie Y Rawsthorne H Tamir D Parker C Breidt F Broadbent J Hutkins R O’Sullivan D Steele J Unlu G Saier M Klaenhammer T Richardson P Kozyavkin S Weimer B Mills D Comparative genomics of the lactic acid bacteria Proc Natl Acad Sci USA 2006 103 15611 15616 10.1073/pnas.0607117103 17030793 PMC1622870

[B10] Mills D Rawsthorne H Parker C Tamir D Makarova K Genomic analysis of PSU-1 and its relevance to winemaking FEMS Microbiol Rev 2005 29 465 475 16125008 10.1016/j.femsre.2005.04.011

[B11] Borneman AR Bartowsky EJ McCarthy J Chambers PJ Genotypic diversity in Oenococcus oeni by high-density microarray comparative genome hybridization and whole genome sequencing Appl Microbiol Biotechnol 2010 86 681 691 10.1007/s00253-009-2425-6 20111862

[B12] Zé-Zé L Tenreiro R Brito L Santos MA Paveia H Physical map of the genome of Oenococcus oeni PSU-1 and localization of genetic markers Microbiology 1998 144 Pt 5 1145 1156 9611789 10.1099/00221287-144-5-1145

[B13] Zé-Zé L Tenreiro R Paveia H The Oenococcus oeni genome: physical and genetic mapping of strain GM and comparison with the genome of a “divergent” strain, PSU-1 Microbiology 2000 146 Pt 12 3195 3204 11101677 10.1099/00221287-146-12-3195

[B14] Bon E Delaherche A Bilhère E De Daruvar A Lonvaud-Funel A Le Marrec C Oenococcus oeni genome plasticity is associated with fitness Appl Environ Microbiol 2009 75 2079 2090 10.1128/AEM.02194-08 19218413 PMC2663225

[B15] Jacobsen A Hendriksen RS Aaresturp FM Ussery DW Friis C The Salmonella enterica pan-genome Microb Ecol 2011 62 487 504 10.1007/s00248-011-9880-1 21643699 PMC3175032

[B16] Lukjancenko O Wassenaar TM Ussery DW Comparison of 61 sequenced Escherichia coli genomes Microb Ecol 2010 60 708 720 10.1007/s00248-010-9717-3 20623278 PMC2974192

[B17] Medini D Donati C Tettelin H Masignani V Rappuoli R The microbial pan-genome Curr Opin Genet Dev 2005 15 589 594 10.1016/j.gde.2005.09.006 16185861

[B18] Vernikos GS Parkhill J Interpolated variable order motifs for identification of horizontally acquired DNA: revisiting the Salmonella pathogenicity islands Bioinformatics 2006 22 2196 2203 10.1093/bioinformatics/btl369 16837528

[B19] El Gharniti F Dols-Lafargue M Bon E Claisse O Miot-Sertier C Lonvaud A Le Marrec C IS30 elements are mediators of genetic diversity in Oenococcus oeni Int J Food Microbiol 2012 158 14 22 10.1016/j.ijfoodmicro.2012.06.009 22809637

[B20] Fujii T Nakashima K Hayashi N Random amplified polymorphic DNA-PCR based cloning of markers to identify the beer-spoilage strains of Lactobacillus brevis, Pediococcus damnosus, Lactobacillus collinoides and Lactobacillus coryniformis J Appl Microbiol 2005 98 1209 1220 10.1111/j.1365-2672.2005.02558.x 15836491

[B21] Suzuki K 125th Anniversary Review: Microbiological instability of beer caused by spoilage bacteria J Instit Brewing 2011 117 131 155 10.1002/j.2050-0416.2011.tb00454.x

[B22] Gindreau E Lonvaud-Funel A Molecular analysis of the region encoding the lytic system from Oenococcus oeni temperate bacteriophage phi 10MC FEMS Microbiol Lett 1999 171 231 238 10077848 10.1111/j.1574-6968.1999.tb13437.x

[B23] Gindreau E Torlois S Lonvaud-Funel A Identification and sequence analysis of the region encoding the site-specific integration system from Leuconostoc oenos (Oenococcus oeni) temperate bacteriophage phi 10MC FEMS Microbiol Lett 1997 147 279 285 10.1016/S0378-1097(96)00540-X 9119205

[B24] Parreira R São-José C Isidro A Domingues S Vieira G Santos MA Gene organization in a central DNA fragment of Oenococcus oeni bacteriophage fOg44 encoding lytic, integrative and non-essential functions Gene 1999 226 83 93 10.1016/S0378-1119(98)00554-X 9889328

[B25] São-José C Santos S Nascimento J Brito-Madurro AG Parreira R Santos MA Diversity in the lysis-integration region of oenophage genomes and evidence for multiple tRNA loci, as targets for prophage integration in Oenococcus oeni Virology 2004 325 82 95 10.1016/j.virol.2004.04.029 15231388

[B26] Bridier J Claisse O Coton M Coton E Lonvaud-Funel A Evidence of distinct populations and specific subpopulations within the species Oenococcus oeni Appl Environ Microbiol 2010 76 7754 7764 10.1128/AEM.01544-10 20935119 PMC2988583

[B27] Borneman AR McCarthy JM Chambers PJ Bartowsky EJ Functional divergence in the genus oenococcus as predicted by genome sequencing of the newly-described species, Oenococcus kitaharae PLoS One 2012 7 e29626 10.1371/journal.pone.0029626 22235313 PMC3250461

[B28] Garvie EI Leuconostoc oenossp.nov J Gen Microbiol 1967 48 431 438 10.1099/00221287-48-3-431 6052633

[B29] Beelman RB Gavin A Keen RM A new strain of Leuconostoc oenos for induced malo-lactic fermentation in Eastern wines Am J Enol Vitic 1977 28 159 165

[B30] Fornachon JCM A Leuconostoc causing malo-lactic fermentation in Australian wines Am J Enol Vitic 1964 15 184 186

[B31] Dimopoulou M Hazo L Dols-Lafargue M Exploration of phenomena contributing to the diversity of Oenococcus oeni exopolysaccharides Int J Food Microbiol 2012 153 114 122 10.1016/j.ijfoodmicro.2011.10.024 22119266

[B32] Bentley SD Aanensen DM Mavroidi A Saunders D Rabbinowitsch E Collins M Donohoe K Harris D Murphy L Quail MA Samuel G Skovsted IC Kaltoft MS Barrell B Reeves PR Parkhill J Spratt BG Genetic analysis of the capsular biosynthetic locus from all 90 pneumococcal serotypes PLoS Genet 2006 2 e31 10.1371/journal.pgen.0020031 16532061 PMC1391919

[B33] Dicks LM Halzapfel WH Vos P, Garrity G, Jones D, Krieg NR, Ludwig W, Rainey FA, Schleifer KH, Whitman WB Genus II. Oenococcus Bergey’s manual of systematic bacteriology 2009 second New York: Springer 635 642

[B34] Barabote RD Saier MH Comparative genomic analyses of the bacterial phosphotransferase system Microbiol Mol Biol Rev 2005 69 608 634 10.1128/MMBR.69.4.608-634.2005 16339738 PMC1306802

[B35] Kelly WJ Asmundson RV Hopcroft DH Growth of Leuconostoc oenos under anaerobic conditions Am J Enol Vitic 1989 40 277 282

[B36] Zavaleta AI Martínez-Murcia AJ Rodríguez-Valera F Intraspecific genetic diversity of Oenococcus oeni as derived from DNA fingerprinting and sequence analyses Appl Environ Microbiol 1997 63 1261 1267 9097422 10.1128/aem.63.4.1261-1267.1997 PMC168419

[B37] Delcher AL Bratke KA Powers EC Salzberg SL Identifying bacterial genes and endosymbiont DNA with Glimmer Bioinformatics 2007 23 673 679 10.1093/bioinformatics/btm009 17237039 PMC2387122

[B38] Altschul SF Madden TL Schäffer AA Zhang J Zhang Z Miller W Lipman DJ Gapped BLAST and PSI-BLAST: a new generation of protein database search programs Nucleic Acids Res 1997 25 3389 3402 10.1093/nar/25.17.3389 9254694 PMC146917

[B39] Bradley RK Roberts A Smoot M Juvekar S Do J Dewey C Holmes I Pachter L Fast statistical alignment PLoS Comput Biol 2009 5 e1000392 10.1371/journal.pcbi.1000392 19478997 PMC2684580

[B40] Guindon S Dufayard J-F Lefort V Anisimova M Hordijk W Gascuel O New algorithms and methods to estimate maximum-likelihood phylogenies: assessing the performance of PhyML 3.0 Syst Biol 2010 59 307 321 10.1093/sysbio/syq010 20525638

[B41] Chen F Mackey AJ Stoeckert CJ Roos DS OrthoMCL-DB: querying a comprehensive multi-species collection of ortholog groups Nucleic Acids Res 2006 34 D363 D368 10.1093/nar/gkj123 16381887 PMC1347485

[B42] Edgar RC MUSCLE: multiple sequence alignment with high accuracy and high throughput Nucleic Acids Res 2004 32 1792 1797 10.1093/nar/gkh340 15034147 PMC390337

[B43] Moriya Y Itoh M Okuda S Yoshizawa AC Kanehisa M KAAS: an automatic genome annotation and pathway reconstruction server Nucleic Acids Res 2007 35 W182 W185 10.1093/nar/gkm321 17526522 PMC1933193

