# Supplementary material for: Comparative analysis of the Oenococcus oeni pan genome reveals genetic diversity in industrially-relevant pathways
Source: BMC Genomics. 2012 Aug 3;13:373. doi: 10.1186/1471-2164-13-373 (PMC3472311; doi:10.1186/1471-2164-13-373)
Supplement: Additional file 4 — Genome-wide results of the “alien hunter” algorithm for each strain. Each individual plot has been spaced according to the whole-genome alignments to ensure that genomic regions are concordant across strains. The area of highest probability of HGT is indicated (black arrow). [file 1471-2164-13-373-S4.pdf]

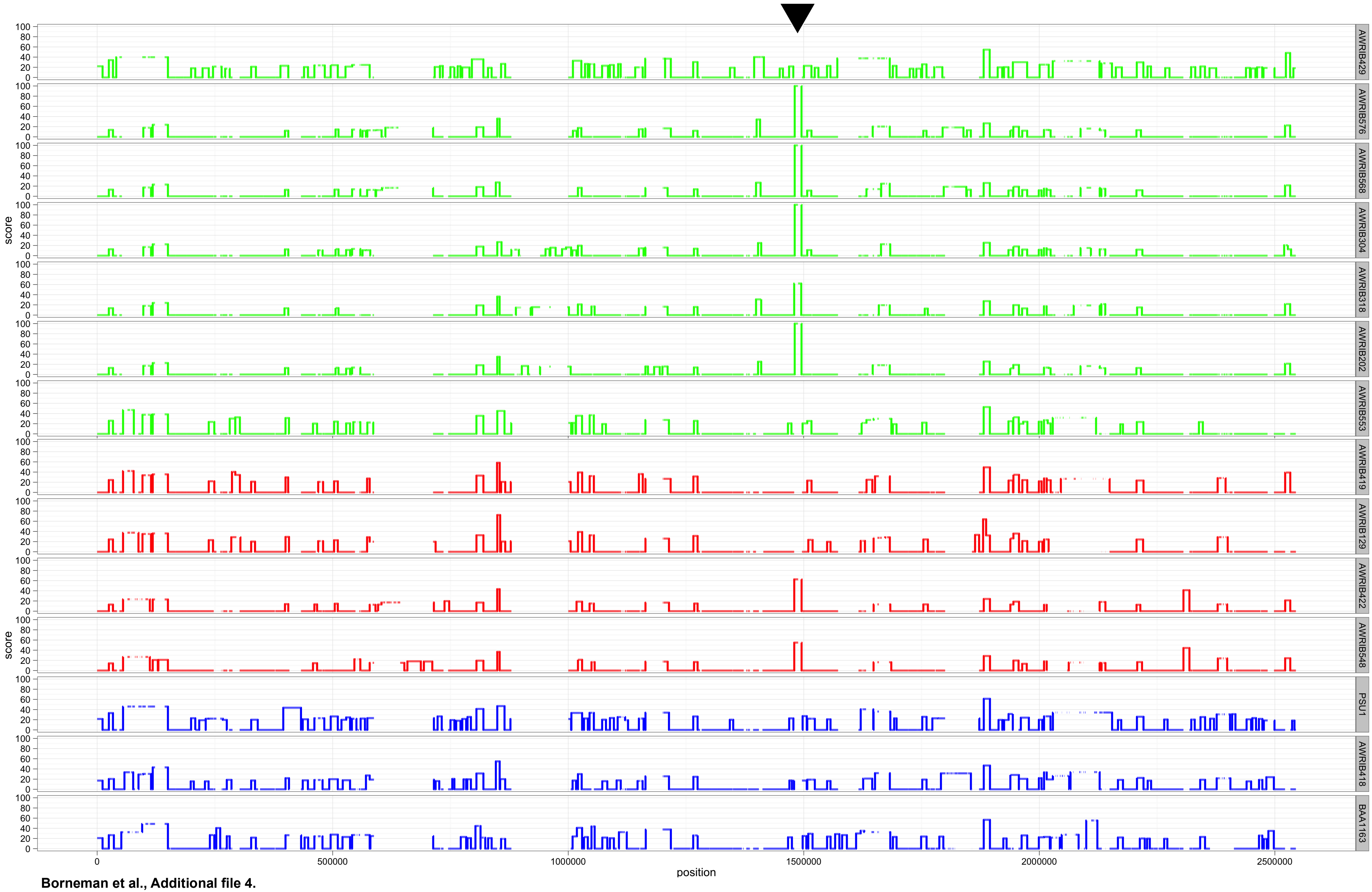

Borneman et al., Additional file 4.

Horizontal transfer of genetic information into the *O. oeni* genome

Genome-wide results of the "alien hunter" algorithm for each strain. Each individual plot has been spaced according to the whole-genome alignments to ensure that genomic regions are concordant across strains. The area of highest probability of HGT is indicated (black arrow).
