# Supplementary figures and images for: Comparative analysis of the Oenococcus oeni pan genome reveals genetic diversity in industrially-relevant pathways
Source: BMC Genomics. 2012 Aug 3;13:373. doi: 10.1186/1471-2164-13-373 (PMC3472311; doi:10.1186/1471-2164-13-373)

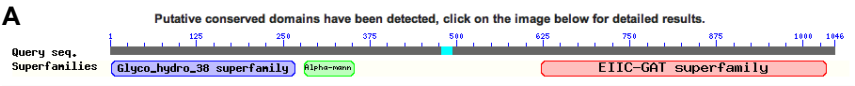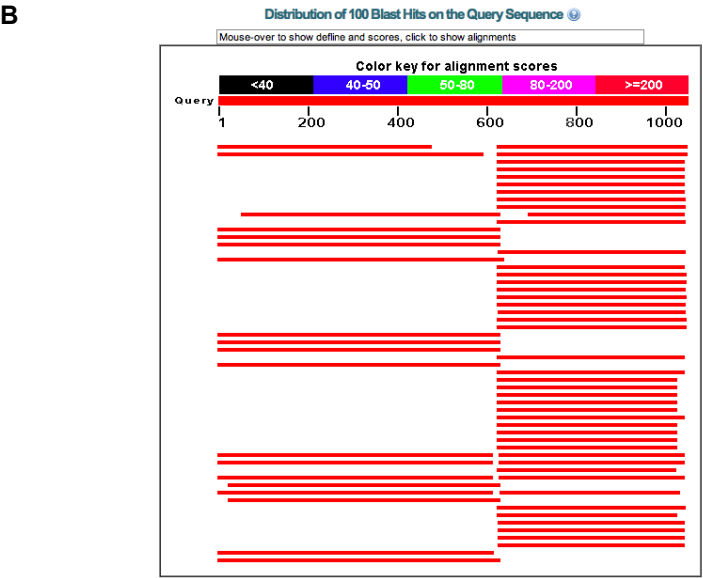

Supplement: Additional file 7 — A novel glycosidase-PTS gene fusion. BLAST homology information for the glycosidase-PTS gene fusion predicted to be encoded by pan_genome ORF 2351. (PDF 44 kb) [file 1471-2164-13-373-S7.pdf]
